# VEGF isoforms have differential effects on permeability of human pulmonary microvascular endothelial cells

**DOI:** 10.1186/s12931-017-0602-1

**Published:** 2017-06-02

**Authors:** Khadija Ourradi, Thomas Blythe, Caroline Jarrett, Shaney L. Barratt, Gavin I. Welsh, Ann B. Millar

**Affiliations:** 1 Academic Respiratory Unit, School of Clinical Sciences, University of Bristol, Bristol, UK; 2 Bristol Renal, School of Clinical Sciences, University of Bristol, Bistol, UK

**Keywords:** Vascular permeability, Vascular endothelial growth factor (VEGF), Cell signalling

## Abstract

**Background:**

Alternative splicing of Vascular endothelial growth factor-A mRNA transcripts (commonly referred as VEGF) leads to the generation of functionally differing isoforms, the relative amounts of which have potentially significant physiological outcomes in conditions such as acute respiratory distress syndrome (ARDS). The effect of such isoforms on pulmonary vascular permeability is unknown. We hypothesised that VEGF_165_a and VEGF_165_b isoforms would have differing effects on pulmonary vascular permeability caused by differential activation of intercellular signal transduction pathways.

**Method:**

To test this hypothesis we investigated the physiological effect of VEGF_165_a and VEGF_165_b on Human Pulmonary Microvascular Endothelial Cell (HPMEC) permeability using three different methods: trans-endothelial electrical resistance (TEER), Electric cell-substrate impedance sensing (ECIS) and FITC-BSA passage. In addition, potential downstream signalling pathways of the VEGF isoforms were investigated by Western blotting and the use of specific signalling inhibitors.

**Results:**

VEGF_165_a increased HPMEC permeability using all three methods (paracellular and transcellular) and led to associated VE-cadherin and actin stress fibre changes. In contrast, VEGF_165_b decreased paracellular permeability and did not induce changes in VE-cadherin cell distribution. Furthermore, VEGF_165_a and VEGF_165_b had differing effects on both the phosphorylation of VEGF receptors and downstream signalling proteins pMEK, p42/44MAPK, p38 MAPK, pAKT and peNOS. Interestingly specific inhibition of the pMEK, p38 MAPK, PI3 kinase and eNOS pathways blocked the effects of both VEGF_165_a and VEGF_165_b on paracellular permeability and the effect of VEGF_165_a on proliferation/migration, suggesting that this difference in cellular response is mediated by an as yet unidentified signalling pathway(s).

**Conclusion:**

This study demonstrates that the novel isoform VEGF_165_a and VEGF_165_b induce differing effects on permeability in pulmonary microvascular endothelial cells.

## Background

VEGF was originally identified by its properties as both a permogen and a mitogen, key elements in the function of the alveolar-capillary membrane, leading to interest in its role in many forms of lung disease particularly ARDS [1–3]. We and others found that VEGF levels were compartmentalised between the alveolar space and the vascular bed [4, 5]. Low levels of intrapulmonary VEGF were found in patients with ARDS with increasing intrapulmonary VEGF levels associated with recovery [5]. In contrast, plasma levels in patients with ARDS were elevated compared with normal, at-risk, or ventilated control subjects, with falling levels associated with recovery [6]. These data suggest that VEGF is beneficial in the alveolar space but detrimental in the vascular space. To explore the significance of these observations, it is necessary to understand the mechanisms that regulate VEGF bioactivity. VEGF exerts its biological effect through specific receptors, VEGF-R1 and VEGF-R2 and co-receptors, neuropilin-1 and neuropilin-2 [7]. In addition, alternative splicing of VEGF transcripts leads to the generation of several functionally different isoforms [8, 9]. We have previously explored changes in VEGF_xxx_-isoforms and receptor expression as mechanisms for regulating VEGF bioactivity and suggested that both these factors may contribute [10] but do not fully explain the reported contradictory findings. The VEGF_xxx_b isoform family consists of peptides of the same length as other forms but with a different C-terminal six amino acids-SLTRKD rather than CDKPRR [11]. The receptor binding and dimerisation domains are intact, but VEGF_xxx_b stimulates a unique pattern of VEGF-R2 tyrosine residue phosphorylation, contrasting with those activated by conventional isoforms [9]. Two specific isoforms, VEGF_165_a and VEGF_165_b isoforms were shown to have contrasting effects on the epithelial and endothelial sides of the alveolar-capillary membrane [12]. These data suggest a pneumotropic effect which could be beneficial within the alveolar space following ARDS. However, the effect of these isoforms on vascular permeability another key element of ARDS is unknown.

We hypothesised that VEGF_165_a and VEGF_165_b activate different signalling pathways mediating cell permeability, a potential explanation for the conflicting observations on effects in the vascular space. To explore this theory, we used three methods of assessing vascular barrier function and found contrasting effects with VEGF_165_a increasing permeability and VEGF_165_b decreasing permeability. We then explored the relationship of downstream pathways to these functional differences. We compared the effects of specific signalling pathway inhibitors of MEK/p38MAPK/PI3K and eNOS on permeability, cell migration and proliferation to identify a mechanism by which increased permeability could be resolved whilst maintaining beneficial cell proliferation and migration.

## Methods

A detailed description of materials and methods is given in the online data supplement.

### Primary cell culture

Human Pulmonary microvascular endothelial cell (HPMEC) cryopreserved from passage 2 (PromoCell, Heidelberg, Germany) were cultured in endothelial cell basal medium MV2 (C-22221, PromoCell, Germany) complemented with supplement pack (C-39221, PromoCell, Germany) according to manufacturer’s instructions.

For all experiments cells were grown to 80% confluence, quiesced (MV2 media only) and stimulated with combinations of VEGF_165_a and VEGF_165_b (20 ng/ml as considered physiologically relevant in circulating plasma) [4, 6] in the presence or absence of specific signalling pathway inhibitors (U0126, SB203580, LY294002 (Cell Signalling, UK) or L-NAME (Calbiochem, UK).

### Measurement of TEER by Endohm

Measurement of trans-endothelial electrical resistance (TEER) of the cell monolayer was performed using an Endohm 12 electrode chamber and an endothelial volt/ohm meter EVOM^2^ (World precision Instruments, USA) as previously described by Bevan and al [13].

### ECIS

Cells were plated at 20000 cells/cm2 into 8-well arrays (8W10E+; Wolf laboratories Ltd). Data was automatically and continuously collected every 2 min and recorded by computer. Experiments were performed after cells reached confluence with basal TEER values > 1500 Hz.

### FITC-BSA passage

Transendothelial permeability to macromolecules was assessed by the passage of FITC-conjugated BSA (relative molecular mass 66,000) across cell monolayers in tissue culture inserts as previously described [14].

### Scratch assay (Migration and proliferation)

Cells were seeded with 100 μl of cell suspension (5 × 10^5^ cells/ml) in an Ibidi culture-chamber (Ibidi GmbH Munich, Germany). Cells were pre-incubated with or without inhibitor for 1 hour before removal of the chamber. Cells were then incubated in MV2 medium alone or MV2 medium with 20 ng/ml of recombinant protein VEGF_165_a or VEGF_165_b. Images were captured and analysed at 0 and 24 h.

### Western blotting analysis

Cell lysates were separated on sodium dodecyl sulphate–polyacrylamide gel electrophoresis (SDS-PAGE) and immunoblotted. Blots were blocked with 5% bovine serum albumin (BSA) (Fischer Scientific UK,) and incubated overnight at 4 °C with primary antibodies

### Immunocytochemistry

HPMEC were stimulated with 100 ng/ml of VEGF_165_a, VEGF_165_b, VEGF_165_a + b or without any stimulation (control) for 10 min. They were then fixed, permeabilised and immunostained for VE-cadherin (Sigma, UK) and Alexa Fluor® 568 Phalloidin (Invitrogen, UK) for staining actin structures.

## Results

### VEGF_165_a increases and VEGF_165_b decreases permeability in HPMEC

**Fig. 1:**
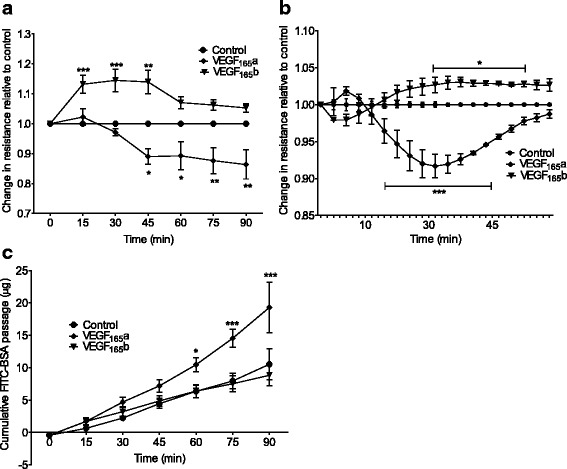
HPMEC stimulated with 20 ng/ml of VEGF_165_a, VEGF_165_b or without any stimulation (control). **a** Paracellular permeability was quantified by TEER using endohm-12 system in HPMEC cultured in inserts. VEGF_165_a reduced resistance (increased permeability) *p < 0.05 (45 min onwards) and VEGF_165_b increased resistance (decreased permeability) ***p < 0.001 (15 to 45 min) compared to control. **b** ECIS measurements on HPMEC show that VEGF_165_a reduced the resistance (increased permeability) significantly***p < 0.001 in comparison to control; whereas VEGF_165_b had an opposite effect with a significant increase (*p < 0.05) in the resistance (reduced permeability). Data expressed as a mean fold-change compared to control over time. **c** The passage of FITC-coupled BSA across the monolayer of HPMEC was monitored over a period of 90 min. The fluorescence intensity of the aliquots was quantified and data was expressed as cumulative FITC-BSA over time. Concentration of FITC-BSA in the lower chamber slowly increased in both control cells and those stimulated with VEGF_165_b; in contrast cells stimulated with VEGF_165_a showed a significant increase in the passage of FITC-BSA *p* < 0.01 after 60 min. Each experiment was performed in triplicate (*n* = 5-8). All data were analysed using two-way ANOVA and Bonferroni post-test for multiple analysis and plotted as mean ± SEM

A number of techniques have been described to assess endothelial barrier function [15]. The barrier properties of endothelial cell junctions can be directly measured by TEER that has been shown to be indirectly correlated with both adherens and tight junctions within a confluent monolayer [16, 17]. We measured the TEER of HPMEC monolayers. VEGF_165_a significantly reduced the cell resistance (increased permeability) **p < 0.01, from 45 min onwards, in contrast, VEGF_165_b significantly increased resistance (decreased permeability) **p < 0.01 between 15 to 45 min compared with unstimulated control cells (Fig. 1a).

ECIS was utilised as another method to evaluate the effect of VEGF isoforms on cell permeability. A similar response to that seen using the Endohm system was observed for HPMEC. VEGF_165_a induced an increase in cell monolayer permeability (***p < 0.001) in contrast to VEGF_165_b which induced a significant decrease (*p < 0.05) in the cell monolayer permeability (Fig. 1b).

The last experimental technique used to assess permeability was FITC-coupled BSA passage. The permeability of the HPMEC monolayer for FITC-BSA was monitored every 15 min up to 90 min by measuring the fluorescence intensity of the medium in the lower compartment. For unstimulated control cells, the level of BSA in the lower compartment increased slowly over time in a comparable manner to those treated with VEGF_165_b; no significant difference was observed between them in HPMEC (Fig. 1c). When the cells were cultured in the presence of VEGF_165_a, a significant (*p < 0.05) time-dependant increase in BSA permeability was detected after 45 min and persisted for at least 90 min, suggesting an increase in the cell monolayer permeability.

### VEGF_165_a induces changes in VE-cadherin distribution pattern and actin stress fibres in HPMEC compared to VEGF_165_b

**Fig. 2:**
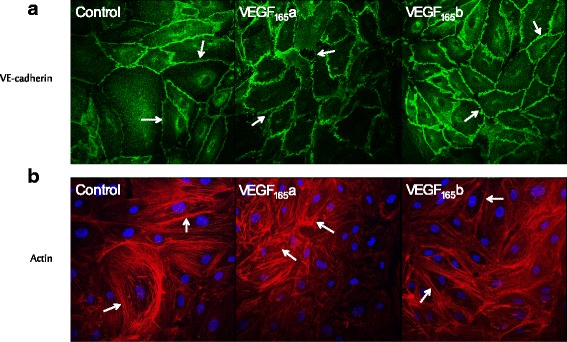
**a** HPMEC were stimulated with 100 ng/ml of VEGF_165_a, VEGF_165_b or without stimulation (control) for 10 min. Then they were fixed, permeabilised and immunostained for VE-cadherin. Control and VEGF_165_b: straight and linear distribution of VE-cadherin at the cell junctions were observed (see *arrows*). VEGF_165_a: distribution of VE-cadherin in a zig-zag pattern with the appearance of gaps between the cells (see *arrows*) (magnification x40). **b** Actin and nucleus staining in HPMEC in different conditions. Control: actin filament distributed mainly at the cell periphery between the cell-cell junctions. VEGF_165_a: disruption of the cortical actin frame with filopodia and stress fibre formation across the cells (*arrows*). VEGF_165_b: mixed field with the appearance of actin at the cell periphery and visible filopodia (magnification x40). (*n* = 5, representative image shown)

Changes in cell permeability are closely related to changes in the cell-cell junction structures [18]. We went on to analyse potential cytoskeleton remodelling induced by VEGF isoforms. VE-cadherin has been particularly associated with endothelial cell adherens junctions but may also contribute to change in tight junction activity [19, 20]. Previously, a changed distribution to a zig-zag pattern in VEGF_165_a treated human umbilical vascular endothelial cells (HUVEC) was demonstrated using 100 ng/ml stimulation [20]. We repeated this experiment using HPMEC and showed similar results suggesting a significant change in VE-cadherin distribution between the cells stimulated with VEGF_165_a (Fig. 2a). Visible gaps appeared between the cells with an apparent zig-zag pattern of VE-cadherin distribution between adjacent cells. In contrast, stimulation with the VEGF_165_b isoform induced minimal change compared to untreated control cells suggesting it did not induce VE-cadherin reorganisation (Fig. 2a). In addition, changes in the actin structure were observed between the stimulated and the unstimulated cells demonstrating an increase of stress fibres across the cells and more actin filopodia with VEGF_165_a. Again, in contrast, there was only a partial induction of actin filaments in the cell periphery and stress fibres by VEGF_165_b (Fig. 2b). These data led to the hypothesis that VEGF_165_a and VEGF_165_b activate different signalling pathways involved in permeability in HPMEC.

VEGF-R2 has been reported to induce most downstream signalling effects through the tyrosine sites tyr1175 and tyr1214 [21, 22]. These two tyrosine sites play a crucial and direct role in the recruitment of adaptor proteins that activate multiple signalling pathways such as proliferation, survival, migration and permeability [23, 24]. Therefore, we studied VEGF isoform induced phosphorylation of those tyrosine sites in HPMECs.

### VEGF_165_a and VEGF_165_b induce differential phosphorylation of the VEGF receptors at tyrosine 1175 and 1214 in HPMEC

**Fig. 3:**
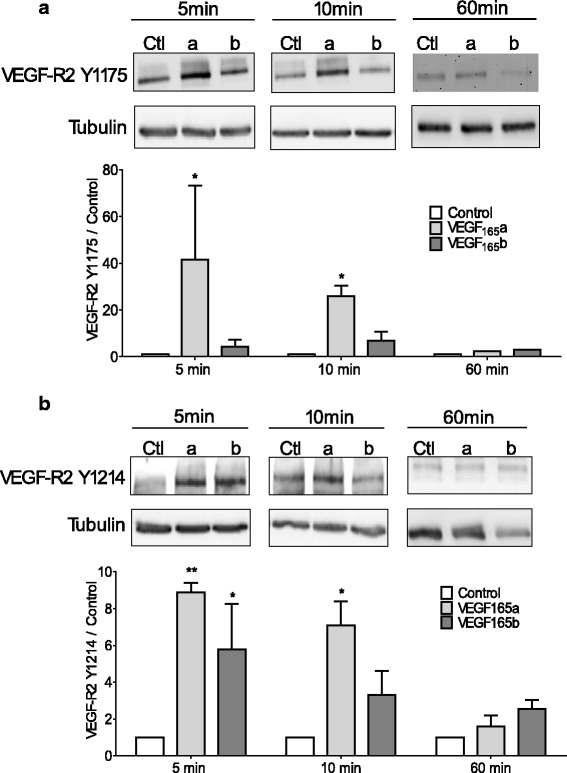
Activation of VEGF-R2 was determined with a phospho-specific antibody recognising tyrosine (Y) site 1175 and site 1214 in graph (**a**) and (**b**) respectively for cells stimulated with 20 ng/ml of VEGF_165_a (**a**), VEGF_165_b (**b**) or without stimulation (Ctl). Representative Western blot showing the different conditions at 5, 10 and 60 min plus the loading control blot and densitometry (*n* = 5). All data were analysed using Kruskal-Wallis with post hoc Dunn’s analysis and plotted as mean ± SEM

VEGF-R2 phosphorylation at the tyrosine (tyr) 1175 site, was significantly induced by VEGF_165_a at 5 min and 10 min (*p* < 0.05) in HPMEC (Fig. 3a) and returned to control levels at 60 min post stimulation. In contrast, VEGF_165_b stimulation did not induce significant phosphorylation at this site. Phosphorylation of VEGF-R2 at the tyr1214 also reached a maximum at 5 min (*p* < 0.01) with VEGF_165_a and subsequently decreased as represented in Fig. 3b. VEGF_165_b also induced significant phosphorylation of tyr1214 site at 5 min (*p* < 0.05) in HPMEC, in contrast to tyr1175. These differences in receptor phosphorylation support the potential for differential binding of other adaptor proteins in addition to the previously reported changes in co-receptor binding that has been shown to contribute to VEGF-induced vascular permeability.

Having identified these phosphorylation differences we wanted to identify specific pathways leading to cell permeability which is less well characterised than those of other VEGF functional effects [7]. Further Western blotting of HPMEC was undertaken, to investigate the effect of VEGF isoforms on the phosphorylation of several proteins previously suggested to be involved in specific functional downstream cellular effects. In parallel, we continued to use the Endohm assay to assess paracellular permeability and utilised the scratch assay combining both migration and proliferation processes [25, 26] to assess the specificity of the functional effect of individual signalling pathway inhibitors.

### Specific inhibition of pMEK1/2, p42/44MAPK and p38MAPK induction by VEGF_165_a and VEGF _165_b in HPMEC does not have a differential effect on permeability and migration/proliferation pathways

The MEK and mitogen activated protein kinase (MAPK) pathways are among the most widely studied in VEGF biology and considered to have critical roles in cell proliferation and cell growth and differentiation [27, 28]. We initially sought to determine whether inhibition of these proteins would have a specific functional effect i.e. inhibit proliferation/migration but not permeability as previously suggested [29].

**Fig. 4:**
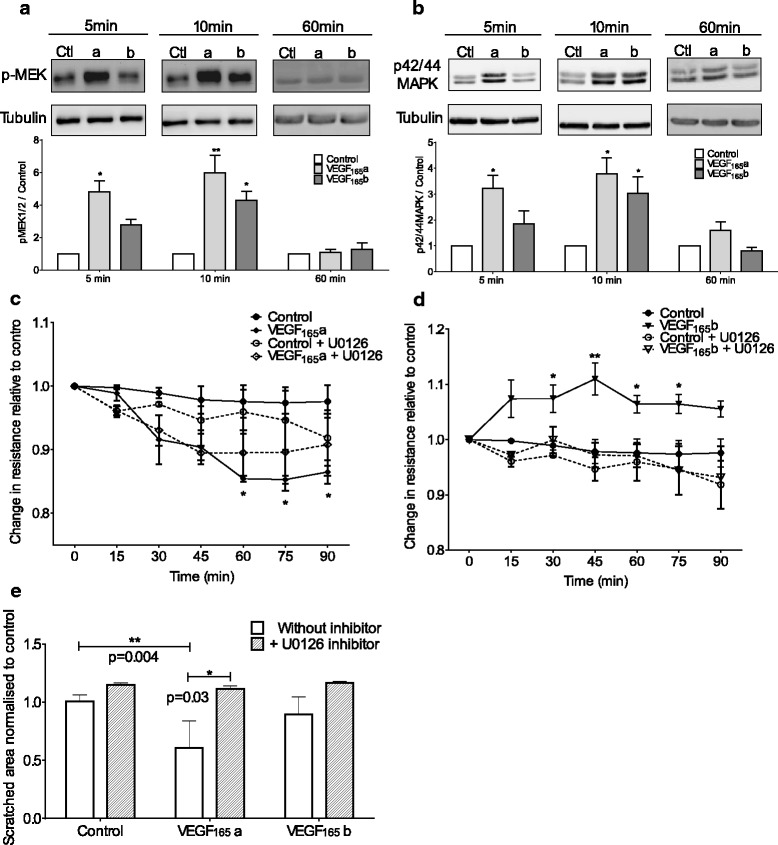
HPMEC were stimulated with 20 ng/ml of VEGF_165_a, VEGF_165_b or without any stimulation (Ctl/control). Representative western blots showing VEGF stimulation at 5, 10 and 60 min plus loading control (α-tubulin) with densitometry (*n* = 3). **a** & **b** Western blot of phosphorylated MEK1/2 (**a**) and phosphorylated p42/p44MAPK (**b**) in HPMEC. Stimulation with VEGF_165_a increased phosphorylation of MEK1/2 and p42/p44 at 5 and 10 min in contrast with VEGF_165_b which induced increased phosphorylation only at 10 min for both proteins. **c** & **d** Measurement of cells resistance by Endohm system. Dotted lines correspond to cell pre-incubated with pMEK inhibitor UO126. UO126 inhibited the effect of VEGF_165_a (**c**) and the effect of VEGF_165_b (**d**). **e** Graphs represent the mean of the scratched area in HPMEC treated with UO126 inhibitor at 24 h relative to control. VEGF_165_a but not VEGF_165_b stimulation significantly diminished scratched area (*p* = 0.004) and was inhibited by UO126 (*p* = 0.03). Densitometry and scratch assay analysed by Kruskal-Wallis test with post hoc Dunn’s analysis. TEER measurement analysed using two-way ANOVA and Bonferroni post-test. All data were plotted as mean ± SEM (*n* = 3-6)

HPMEC showed a similar activation pattern of pMEK1/2 and p42/44MAPK proteins with an increase in the phosphorylation when treated with VEGF_165_a for 5 and 10 min that subsequently returned towards baseline at 60 min (Fig. 4-a&b respectively). VEGF_165_b had a delayed effect with significant phosphorylation of both proteins occurring at 10 min. The pMEK1/2 phosphorylation in response to VEGF isoforms showed a significant increase at 5 and 10 min (*p* < 0.01) for the cells stimulated with VEGF_165_a compared to control. The maximum response was observed at 10 min for both proteins. However, VEGF_165_b induced a significant increase of pMEK1/2 and p42/44MAPK phosphorylation at 10 min only. At 60 min the level of both phospho-proteins in the stimulated cells returns toward the control levels.

U0126 is a compound reported to be a highly selective inhibitor of MEK1 and MEK2 and utilised to block the classical MAPK cascade in cells that leads to cell proliferation [30]. U0126 inhibited the permeability effects of both VEGF_165_a and VEGF_165_b on HPMEC (Fig. 4c and d respectively). VEGF_165_a, but not VEGF_165_b, induced a significant increase in the cell migration into the “scratch” (*p* = 0.004) in comparison to control (Fig. 4e) that was inhibited byU0126. These data suggest that the MEK pathway is activated by both VEGF_165_a and VEGF _165_b but any functional divergence must occur downstream.

**Fig. 5:**
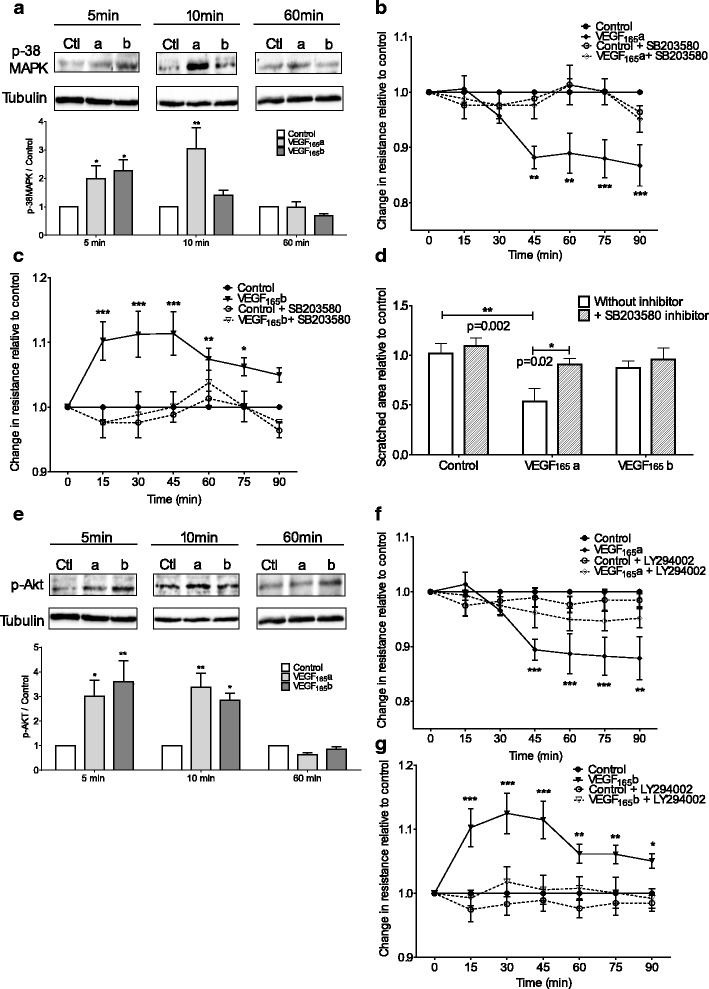
HPMEC were stimulated with 20 ng/ml of VEGF_165_a, VEGF_165_b or without stimulation (Ctl/control). Representative western blots showing VEGF stimulation at 5, 10 and 60 min plus loading control (α-tubulin) with densitometry (*n* = 3). **a** Western blot of phosphorylated p-38MAPK in HPMEC. VEGF_165_a significantly increased phosphorylation of p-38MAPK at 5 and 10 min where VEGF_165_b only induced increased phosphorylation at 5 min. **b** & **c**: Measurement of HPMEC resistance by Endohm system. Dotted lines correspond to cell pre-incubated with p38 inhibitor SB203580. SB203580 inhibited the effect of VEGF_165_a (**b**) and the effect of VEGF_165_b (**c**). **d** Graphs represent the mean of the scratched area for HPMEC treated with SB203580 inhibitor at 24 h relative to control. VEGF_165_a but not VEGF_165_b stimulation significantly diminished scratched area (*p* = 0.002) and was inhibited by SB203580 (*p* = 0.02). **e** Western blot of phosphorylated AKT in HPMEC. VEGF_165_a and VEGF_165_b significantly increased phosphorylation of p-Akt at 5 and 10 min. **f** & **g** Measurement of HPMEC resistance by Endohm system. Dotted lines correspond to cell pre-incubated with AKT inhibitor LY294002. LY294002 inhibited the effect of VEGF_165_a (**f**) and the effect of VEGF_165_b (**g**). Densitometry and scratch assay analysed by Kruskal-Wallis test with post hoc Dunn’s analysis. TEER measurement analysed using two-way ANOVA and Bonferroni post-test. All data were plotted as mean ± SEM (*n* = 3-6)

In addition, phosphorylation of p38MAPK was assessed as studies reported that VEGF stimulation on endothelial cells, also lead to activation of the Cdc42/p38MAPK pathway which triggers cytoskeletal modifications [31]. Similarly, to the other proteins studied, p38 MAPK phosphorylation was significantly increased by VEGF_165_a in HPMEC at 5 and 10 min (*p* < 0.05). Cells stimulated with VEGF_165_b showed a significant increase in p38MAPK phosphorylation (*p* = 0.04) compared to the untreated cells (Fig. 5a). Nevertheless, this phosphorylation was transient and returned to baseline levels by ten minutes. SB203580 is reported to specifically inhibit the activation of SAPK2/p38 MAPK in cell-based assays but no other related kinase protein including MAPK family members [30]. When HPMEC were incubated with SB203580 (p38MAPK inhibitor) again there was inhibition of VEGF_165_a and VEGF_165_b permeability effects (Fig. 5b and c). Cell proliferation/migration into the “scratch” was significantly increased (*p* = 0.002) only with VEGF_165_a stimulation and this effect was inhibited by SB203580 (Fig. 5d).

Having identified significant differences in the activation kinetics of MEK, p42/44MAPK and p38MAPK by VEGF_165_a compared to VEGF_165_b (that reflected changes in permeability but not specific to it alone), we then looked at the effect of VEGF isoforms on AKT also known as protein kinase B (PKB) protein. AKT has been shown to promote the proposed cell survival pathway mediated by the activation of PI3-kinase protein kinase [32] but it has been also associated with eNOS production which is closely associated with permeability [33].

### Specific inhibition of AKT induction by VEGF_165_a and VEGF_165_b in HPMEC does not have a differential effect on permeability and survival pathways

VEGF_165_b induces a rapid and robust phosphorylation of pAKT at 5 min and 10 min (*p* < 0.01) in HPMEC with maximal AKT phosphorylation detected at 5 min (Fig. 5e). Stimulation with VEGF_165_a also induced a significant phosphorylation of AKT at 5 (*p* < 0.05) and 10 min (*p* < 0.01) and returned to control levels at 60 min. LY294002 compounds is an inhibitor of phosphatidylinositol 3 kinase (PI3K) and inactivation of PI3K have been reported to lead to dephosphorylation of Akt that subsequently stop G1 cycle in cell growth and ultimately lead to cell apoptosis [34, 35]. Co-culture with LY294002 to block AKT protein inhibited both the permeability effects of VEGF_165_a and VEGF_165_b on HPMEC (Fig. 5 f and g). In contrast with the permeability assay where the cell monolayer was stable over the experiment (2 h), inhibition of AKT for long periods (24 h) induced cell death so we were unable to undertake the scratch assay as a migration/proliferation model.

Finally, we investigated the potential of the eNOS pathway for differential effects. There is a significant body of evidence to suggest that endothelial nitric oxide (synthesised by eNOS) may have a crucial role in causing hyperpermeability in response to pro-inflammatory agents such as VEGF [36, 37]. Therefore, the effects of the different VEGF isoforms on the eNOS phosphorylation in HPMEC were studied.

### Specific inhibition of eNOS induction by VEGF_165_a and VEGF_165_b in HPMEC does not have a differential effect on permeability and nitric oxide signalling pathways

**Fig. 6:**
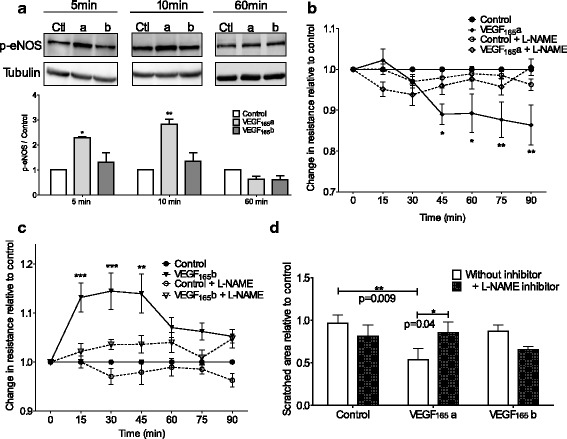
**a** Representative Western blot of phosphorylated p-eNOS in HPMEC (*n* = 3). VEGF_165_a significantly increased phosphorylation of eNOS at 5 and 10 min in contrast with VEGF_165_b stimulation which did not induce any changes. **b** & **c** Measurement of HPMEC resistance by Endohm system. Dotted lines correspond to cell pre-incubated with inhibitor. L-NAME inhibited the effect of VEGF_165_a (**b**) and the effect of VEGF_165_b (**c**). **d** Graphs represent the mean of the scratched area for HPMEC treated with L-NAME inhibitor at 24 h relative to control. VEGF_165_a but not VEGF_165_b stimulation significantly diminished scratched area (*p* = 0.009) and was inhibited by L-NAME (*p* = 0.04). Densitometry and scratch assay were analysed by Kruskal-Wallis test with post hoc Dunn’s analysis. TEER measurement data were analysed using two-way ANOVA and Bonferroni post-test for multiple analysis. All data were plotted as mean ± SEM (*n* = 3-6)

In HPMEC, VEGF_165_a induced a significant increase in the phosphorylation of eNOS at 5 (*p* < 0.05) and 10 min (*p* < 0.01) compared to untreated control cells (Fig. 6a). VEGF_165_b did not induce changes in the phosphorylation of eNOS at Ser 1177. L-NAME (NG-Nitro-L-arginine Methyl Ester, Hydrochloride) is an analogue of L-arginine required for the nitric oxide synthesis by the vascular endothelium [38]. HPMEC pre-incubated with L-NAME and treated with VEGF_165_a or VEGF_165_b did not have any significant change in resistance compared to control (Fig. 6b and c). In the scratch proliferation/migration model, L-NAME inhibited the migration effect induced by VEGF_165_a, but no difference was observed for cells stimulated by VEGF_165_b (Fig. 6d). The inhibition of those effects induced by both VEGF_165_a and VEGF_165_b by L-NAME suggests that eNOS is not involved in the differential effect on permeability induced by these VEGF isoforms.

## Discussion

The pulmonary endothelium is crucial to the regulation of the passage of solutes and molecules between the blood and the interstitial space of the lung, enabling close proximity of the vascular bed to the alveolar space for gaseous exchange to occur. Despite this, there is a very limited understanding of the mechanisms involved in the regulation of pulmonary endothelial cell (EC) barrier function integrity, which is so essential for maintaining this critical function of the lung.

Vascular endothelial growth factor (VEGF) was originally described as both an angiogenic and a permeability factor [39] and its effects have previously been studied using the human umbilical vein endothelial cell (HUVEC) as the archetypal EC. Large organ functional differences are reflected in the variability of endothelial cell junction structure and composition particularly relevant in the functional differences between the pulmonary and systemic circulation [40, 41]. To explore our hypothesis and its relationship to previous clinical studies (5, 6) it was important to study the response of human pulmonary microvascular endothelial cell (HPMEC) to VEGF isoforms.

Among all the pathological processes involved in. ARDS increases in lung vessel permeability are critical and non-redundant [42]. The measurement of permeability in this study has been undertaken only in-vitro models with self-evident limitations [43]. Transport of plasma proteins, cell and solutes across monolayers occurs paracellularly via specialised endothelial cell-cell junctions, or transcellularly by special transport mechanism including transcytosis, via transcellular channels or cell membrane transporter proteins.

Two types of inter-endothelial junction are present in the endothelium, adherens and tight junctions, the former being dominant in most vascular beds. The integrity of the adherens junction is particularly critical for regulating paracellular permeability via homophilic adhesions between VE-cadherin molecules [19, 20]. Disruptions of these domains lead to downstream events that result in organisational changes in the actin cytoskeleton [44]. The transcellular pathway is responsible for the transport of larger molecules such as albumin across endothelial cell monolayers, classically via transcellular pores associated with caveolae and lipid rafts [45]. Traditionally these pathways have been considered independent but there is now a body of evidence showing interdependence [46]. Following on from this, the in-vitro methods of measuring permeability that we have used, TEER (thought to reflect only paracellular permeability) and FITC-BSA (thought to only reflect transcellular mechanism) are recognised to have influences from crosstalk between both pathways [15].

We have demonstrated for the first time that VEGF_165_a and VEGF_165_b induce differing effects on the permeability of pulmonary microvascular endothelial cells. Specifically, VEGF_165_a induced an increase and VEGF_165_b a decrease in permeability. The receptor binding and dimerisation domains are intact in the VEGF_xxx_b family of VEGF isoforms. However, in porcine aortic endothelial cells, VEGF_165_b has been shown to stimulate a unique pattern of VEGF-R2 tyrosine residue phosphorylation, contrasting with those activated by conventional isoforms suggesting activation of differing downstream signalling pathways in addition to partial agonist activity and changes in neuroplin-1 binding [22, 47, 48]. In this study, differing phosphorylation kinetics were clearly observed following stimulation by VEGF_165_a and VEGF_165_b using what we considered to be physiologically relevant concentrations of VEGF.

The differential effects of VEGF_165_a and VEGF_165_b on the vascular permeability in addition to those we have previously shown and published on proliferation in HPMEC (also repeated in the scratch experiments) and human alveolar epithelial cells offer a potential paradigm to explain the apparent compartmentalisation of VEGF between the alveolar and vascular space and the apparent disparity of data relating to the role of VEGF in ARDS [5, 6, 12].

We identified that VEGF_165_a and VEGF_165_b lead to differential functional outcomes with VEGF_165_a increasing cell permeability in methods suggested to reflect both para and transcellular permeability and VEGF_165_b reducing paracellular permeability only. This suggested that the differences were due to divergence of signalling pathways and therefore potential targets for amelioration of outcome e.g. reducing permeability whilst preserving a pneumotropic effect. To verify this hypothesis, different protein inhibitors have been used to look at their effect on the change in resistance reflecting the paracellular permeability pathway of pulmonary microvascular ECs.

The VEGF_165_a signalling pathways have been studied extensively in HUVEC although studies in HPMEC are limited [41]. We chose to use inhibitors of pMEK, P38 MAPK, PI3 kinase and eNOS proteins as these proteins have been suggested to be involved in VEGF signalling pathways and look at both permeability and proliferation/migration in an attempt to identify a divergence in functionality and thus an opportunity for selective inhibition.

The use of L-NAME (eNOS inhibitor) and LY294002 (PI3K inhibitor) on HPMEC inhibited the effect of both VEGF_165_a and VEGF_165_b. Being part of the same signalling pathway these results suggest that VEGF cell paracellular permeability involves the phosphoinositide 3-kinase–AKT pathway, which then further phosphorylates and activates endothelial nitric oxide synthase (eNOS) [49]. Also, the inhibition of pMEK and p38 MAPK did not affect VEGF isoforms activity on the proliferation and paracellular permeability pathway in HPMEC. In summary, the inhibitors chosen did not allow for the identification of specific differential pathways between VEGF_165_a and VEGF_165_b. Further studies of other pathways are required in order to unravel the molecules responsible for the differential permeability effects of VEGF isoforms such as Src kinase pathway and its role in the regulation of endothelial-barrier integrity as demonstrated recently by Gao et al. [50].

## Conclusion

HPMEC have specific signalling characteristics that are probably adaptations to the unique pulmonary environment. In the context of ARDS, VEGF (from the alveolus) may be a triggering component leading to further endothelial dysfunction and failure of the ACM. The data presented has shown that exposure of HPMEC to exogenous VEGF_165_a significantly increased endothelial cell monolayer permeability which would lead to failure of the ACM barrier function, of particular significance in the lung environment. On the other hand, depending on the isoforms present, VEGF may also be a protective agent, the data presented showing a decrease in permeability with VEGF_165_b exposure. Further work will need to be undertaken to clearly identify the divergence in the permeability signalling pathway induced by both isoforms and the potential protective proprieties of VEGF_165_b.

## Abbreviations

°C: Degrees celsius
ACM: Alveolar capillary membrane
AKT: AKT8 virus oncogene cellular homolog
ANOVA: Analysis of variance
ARDS: Acute respiratory distress syndrome
BSA: Bovine serum albumin
Ctl: Control
DAPI: 4′,6-diamidino-2-phenylindole
EC: Endothelial cell
ECIS: Electrical cell impedance system
eNOS: Endothelial nitric oxide synthase
EVOM: Epithelial voltohmmeter
FITC: Fluorescein isothiocyanate
HPMEC: Human pulmonary microvascular endothelial cell
HUVEC: Human umbilical vein endothelial cell
L-NAME: NG-Nitro-L-arginine Methyl Ester
MAPK: Mitogen-activated protein kinase
MEK: Mitogen/extracellular signal-regulated kinase
Min: Minutes
p38MAPK: p38 mitogen-activated protein kinase
PI3K: Phosphoinositide 3-kinase
SDS-PAGE: Sodium dodecyl sulphate polyacrylamide gel electrophoresis
SEM: Standard error of the mean
SRC: V-scr sarcoma (Schimidt-Ruppin A-2) viral oncogene homolog
TEER: Transendothelial electrical resistance
Tyr: Tyrosin
UK: United Kingdom
USA: United States of America
VE-Cadherin: Vascular endothelial cadherin
VEGF: Vascular endothelial growth factor
VEGF-R1: Vascular endothelial growth factor –receptor 1
VEGF-R2: Vascular endothelial growth factor –receptor 2
WB: Western blotting
